# CD164 regulates the tumorigenesis of ovarian surface epithelial cells through the SDF-1α/CXCR4 axis

**DOI:** 10.1186/1476-4598-12-115

**Published:** 2013-10-05

**Authors:** Ai-Fang Huang, Min-Wei Chen, Shih-Ming Huang, Chu-Lien Kao, Hung-Cheng Lai, James Yi-Hsin Chan

**Affiliations:** 1 Graduate Institute of Medical Sciences, National Defense Medical Center, Taipei 114, Taiwan, Republic of China; 2 Departments of Oncology and Pathology, National Taiwan University Hospital, Taipei 100, Taiwan, Republic of China; 3 Department of Biochemistry, National Defense Medical Center, Taipei 114, Taiwan, Republic of China; 4 Department of Microbiology and Immunology, National Defense Medical Center, Taipei 114, Taiwan, Republic of China; 5 Department of Obstetrics and Gynecology, Tri-Service General Hospital, National Defense Medical Center, Taipei 114, Taiwan, Republic of China; 6 Department of Family and Community Medicine, Tri-Service General Hospital, National Defense Medical Center, Taipei 114, Taiwan, Republic of China

**Keywords:** Ovarian cancer, CD164, CXCR4, SDF-1α, Tumorigenesis

## Abstract

**Background:**

CD164 (endolyn), a sialomucin, has been reported to play a role in the proliferation, adhesion, and differentiation of hematopoietic stem cells. The potential association of CD164 with tumorigenicity remains unclear.

**Methods:**

The clinicopathological correlation of ovarian cancer with CD164 was assessed in a 97-patient tumor tissue microarray. Overexpression or silence CD164 was to analyze the effect of CD164 on the proliferation, colony formation and apoptosis via a mouse xenograft and western blotting analysis. The subcellular localization of CD164 was collected in the immunohistochemical and confocal analysis.

**Results:**

Our data demonstrated that higher expression levels of CD164 were identified in malignant ovarian cancer cell lines, such as SKOV3 and HeyA8. The clinicopathological correlation analysis showed that the upregulation of CD164 protein was significantly associated with tumor grade and metastasis. The overexpression of CD164 in human ovarian epithelial surface cells promoted cellular proliferation and colony formation and suppressed apoptosis. These tumorigenicity effects of CD164 were reconfirmed in a mouse xenograft model. We also found that the overexpression of CD164 proteins increased the amounts of CXCR4 and SDF-1α and activated the SDF-1α/CXCR4 axis, inducing colony and sphere formation. Finally, we identified the subcellular localization of CD164 in the nucleus and cytosol and found that nuclear CD164 might be involved in the regulation of the activity of the CXCR4 promoter.

**Conclusions:**

Our findings suggest that the increased expression of CD164 is involved in ovarian cancer progression via the SDF-1α/CXCR4 axis, which promotes tumorigenicity. Thus, targeting CD164 may serve as a potential ovarian cancer biomarker, and targeting CD164 may serve as a therapeutic modality in the management of high-grade ovarian tumors.

## Introduction

Ovarian carcinomas are among the most lethal gynecological malignancies for women in the world [1,2]. Despite advances in treatment, including surgery and targeted chemotherapy, the 5-year survival of patients with epithelial ovarian cancer (EOC) remains only 45% [3]. The poor ratio of survival to incidence in EOC patients results from the high percentage of cases diagnosed at an advanced stage. Although some studies have shown that the fallopian tube could be a source of ovarian cancer, most of the ovarian cancers are classified as “epithelial” and are believed to arise from the surface of the ovary [4,5]. Ovarian carcinomas are highly metastatic tumors that primarily invade the surrounding tissues and serosal cavities, and their spread through the systemic circulation is uncommon [6]. Although many previous studies have focused on the mechanisms underlying the development and progression of ovarian cancer [7-10], the actual series of events of ovarian cancer tumorigenesis are not yet clear.

CD164 (also known as endolyn) is a glycoprotein and a type I integral transmembrane sialomucin. Studies have shown that CD164 may serve as a signaling receptor that regulates proliferation, adhesion and migration in hematopoietic stem and progenitor cells [11,12]. CD164 is also involved in the development and regeneration of skeletal muscle [13]. Furthermore, CD164 serves as a factor in the regulation of prostate cancer cell adhesion to the human bone marrow endothelial monolayer [14]. Recently, Li *et al.* reported that the mobility and metastasis of colon cancer cells were decreased while CD164 expression was knocked down, suggesting that CD164 may play an important role in colon cancer progression [15]. An earlier study showed that CD164 acts as a component of a CXCR4 complex and regulates the SDF-1α-mediated migration of CD133^+^ cells [11]. SDF-1α enhances the mRNA expression of CD164 and alters the protein expression of CD164 [14]. The CXCR4 chemokine receptor has been implicated in many malignancies [14,15], and the SDF-1α/CXCR4 axis has been shown to be involved in several aspects of tumor progression, including angiogenesis, metastasis and survival [16-20]. CD164 associates with the chemokine receptor CXCR4 [13], possibly as a co-receptor for the CXCR4 ligand SDF-1α. These results reveal that CD164 may be the key molecule in the modulation of the tumor progression.

In this study, the CD164 expression profiles of ovarian cancer cells were measured and were suggested to have a correlation with ovarian tumorigenesis processes, including proliferation, migration and invasion. CD164 in human ovarian surface epithelial cells was overexpressed to study the functional roles of CD164 and revealed that CD164 might modulate the SDF-1α/CXCR4 axis to promote ovarian tumorigenesis via the induction of SDF-1α and CXCR4. In summary, our work opens the door to studying the functions of CD164 in tumorigenesis as well as in stem cell differentiation.

## Results

### CD164 is highly expressed in ovarian cancer cell lines and tissues and serves as a prognostic marker

**Figure 1:**
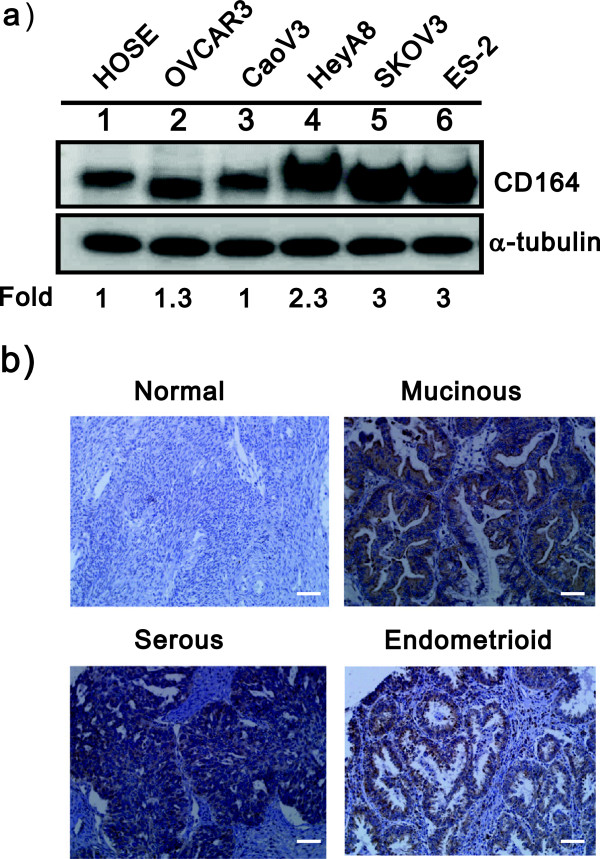
**Quantification of the expression of CD164 in ovarian cancer cell lines and an ovarian tissue array. a)** Cell lysates from indicated ovarian cells were subjected to immunoblot analysis with an antibody against CD164 protein. α-tubulin was used as a loading control. **b)** Immunohistochemical staining of malignant and normal ovarian tissue section stained with rabbit anti-CD164 antibodies and CD164 levels in representative ovarian tumor tissue. Scale bars = 50 μm.

To address whether CD164 is involved in ovarian tumorigenesis, we measured the expression of CD164 in some ovarian cancer cell lines and the normal ovarian cell line, hOSE, by immunoblotting analysis. As shown in Figure 1a, the highly invasive cell lines, HeyA8, SKOV3 and ES-2 cells, expressed higher levels of CD164 compared to the less malignant cell lines, OVCAR3 and Caov3, and the hOSE cells. To determine the association between the abundance of the CD164 protein and ovarian cancer, we used a tissue microarray containing normal ovarian tissue, benign tumor tissue and different stages of malignant tumors for immunohistochemical staining. The CD164 staining localized to both the cytoplasm and the cell membrane, and most tumors were strongly stained in their nuclei and had a uniform staining pattern in the epithelial component but not in the stroma (Figure 1b). Furthermore, tissues from different stages of ovarian cancers were stained to determine the amount of CD164 protein (+1 faint, +2 moderate, +3 strong and +4 very strong). Our findings indicated that a high abundance of CD164 protein was only significantly correlated with high-grade ovarian tumors (P < 0.001) (Table 1**)**. Hence, the expression level of the CD164 protein could be used as a prognostic marker for ovarian cancer.

**Table 1:** Clinicopathological analysis of CD164 gene expression in an ovarian cancer tissue array

**Characteristics**	**Total**	**< = 3-fold**	**>3-fold**	**p-value**
**All cases**	**97**	**25 (25.8%)**	**72 (74.2%)**	
** Age**				
<50 yrs	**39**	**23 (58.9%)**	**16 (14.1%)**	
≥50 yrs	**58**	**34 (58.6%)**	**24 (41.4%)**	**0.972**
** Stage**				
Early (T1 + T2)	**72**	**51 (70.9%)**	**21 (29.1)**	
Late (T3)	**25**	**5 (20.0%)**	**34 (87%)**	**< 0.05**
** Grade**				
Low (1 & 2)	**50**	**36 (72.0%)**	**14 (28.0%)**	
High (3)	**46**	**5 (10.9%)**	**41 (89.1%)**	**< 0.001**
** Histology**				
Serous	**49**	**30 (61.2%)**	**19 (38.8%)**	
Others	**48**	**34 (71.0%)**	**14 (29.0%)**	**0.317**
** Metastasis**				
Absent	**73**	**46 (63.1%)**	**27 (36.9%)**	
Present	**24**	**8 (33.4%)**	**16 (66.7%)**	**< 0.05**

### Overexpression of CD164 alters cell morphology and induces malignant transformation/anchorage independent growth in hOSE cells

**Figure 2:**
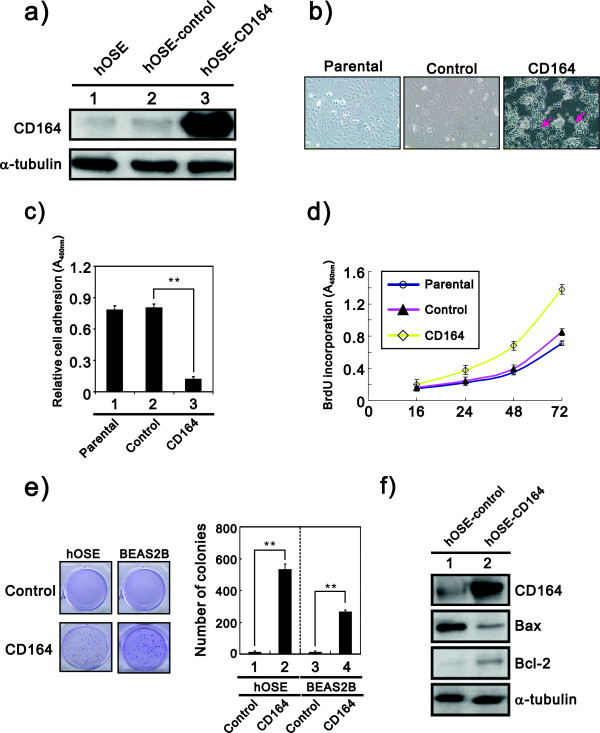
**CD164 overexpression alters cell morphology and induces malignant transformation/anchorage independent growth in hOSE. a)** Cell lysates from hOSE-CD164 cells were subjected to an immunoblot analysis with an antibody against the CD164 protein. α-tubulin was used as a loading control. **b)** The morphology of hOSE-CD164 cells compared with hOSE parental and hOSE-vector control cells by phase-contrast microscopy. (×100 magnification and Scale bars = 50 μm). **c)** The adhesive ability of hOSE-CD164 and control cells was detected by an adhesion assay. **p < 0.01 (three independent experiments were performed and the data are expressed as the means ± s.e.m.). **d)** Proliferation of hOSE-CD164 cells and control cells were analyzed using a BrdU proliferative assay (three independent experiments and data were mean ± s.e.m.). **e)** Effect of CD164 overexpression on the colony formation ability of hOSE cells. Soft agar colony formation of hOSE-CD164 and BEAS2B-CD164 cells paired with control cells was calculated after 28 days of culture. The graph showed the number of colonies (means ± s.e.m.) after 4 weeks of culture for three independent experiments. The *p* values (determined by Student’s *t* test) were relative to control cells. **p < 0.01 (three independent experiments and data were means ± s.e.m.). **f)** Cell lysates from hOSE-CD164 cells were subjected to immunoblot analysis for antibodies against anti-apoptotic Bcl-2 and apoptotic Bax protein. α-tubulin was used as a loading control.

To examine the transforming effect of CD164 in non-cancer cells, such as hOSE, we overexpressed the CD164 gene (Figure 2a) and evaluated the effect of its overexpression on the cell morphology, proliferation, adhesion and anchorage-independent colony formation. The effect of CD164 overexpression on cellular morphology was examined with phase-contrast microcopy, and the CD164-overexpressing hOSE cells showed fewer cell-cell contacts and exhibited scattering of cells (Figure 2b). Furthermore, the CD164-overexpressing cells had lower adhesion ability than the hOSE-vector control cells (Figure 2c). Additionally, the cell proliferation rate was **s**ignificantly increased in the CD164-overexpressing hOSE cells in comparison to the vector-control cells via the BrdU proliferation assay **(**Figure 2d). In addition, the effect of CD164 on anchorage independent growth was determined by the colony numbers and sizes after 4 weeks of growth in soft agar. The hOSE-CD164 cells formed many more colonies than the hOSE-vector control cells using an anchorage-independent colony formation assay (Figure 2e). Interestingly, we also observed a higher expression of the anti-apoptotic protein Bcl-2 and a lower expression of the apoptotic protein Bax in CD164-overexpressed hOSE cells (Figure 2f). These results suggested that CD164 modulates tumor progression by increasing proliferation and anchorage-independent growth through anti-apoptotic effects, and therefore, it might play a role in the promotion of the tumorigenic potential of ovarian surface epithelial cells.

### CD164 overexpression promotes ovarian tumor formation

**Figure 3:**
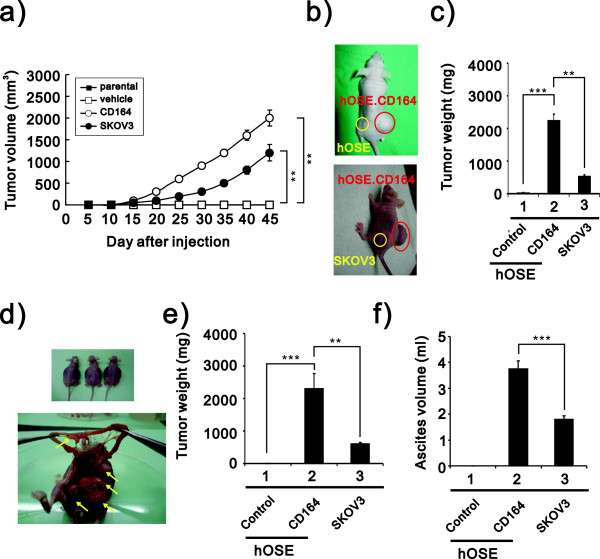
**CD164 overexpression promotes tumor formation in nude mice**. **a)** The tumor growth of hOSE-CD164 cells, control cells and SKOV3 cells subcutaneously injected into female athymic nude mice (*n = 8*, each group) was assessed every 5 days for 45 days by measuring two perpendicular diameters and calculating the tumor volume in mm^3^. Tumor size was measured during 7–8 weeks and calculated as follows: volume = length × width^2^ × 1/2. ** *p* < 0.01. Data represent the means ± SEM. **b)** Macroscopic appearance of hOSE-CD164 (top and bottom, red), hOSE control (top, yellow) and SKOV3 (bottom, yellow) tumors formed subcutaneous tumors. **c)** Quantification of the tumor weight of hOSE-vector and hOSE-CD164 cells (4 × 10^6^) after subcutaneous injection in the flanks of nude mice (*n = 8*, each group). Tumor weight was measured in the hOSE-CD164 cells and compared with hOSE-vector cells (*** *p* < 0.001) and SKOV3 (** *p* < 0.01) nude mice. **d)** Macroscopic appearance of hOSE-CD164 tumors (upper) spread and disseminated in the peritoneal cavity (bottom, arrows). **e)** hOSE-CD164 cells, control cells and SKOV3 cells (4 × 10^6^) were injected into the peritoneal cavity of nude mice. Tumor weight was measured in the hOSE-CD164 cells and compared with hOSE-vector cells (*** *p* < 0.001) or SKOV3 (** *p* < 0.01) nude mice. **f)** At autopsy, tumors were excised and ascite fluid was collected and measured. *** *p* < 0.001.

Based upon these observations, we hypothesized that CD164 plays important roles in ovarian tumor growth *in vivo*. Because generating tumors by subcutaneous injection allows for easy monitoring of tumor growth, and intra-peritoneal injection allows tumor cells to grow in a peritoneal microenvironment that mimics the microenvironment of ovarian cancer tumors, we developed two xenograft models in nude mice to evaluate the tumor formation ability of the hOSE-CD164 and SKOV3 cells (as a positive control). After 8 weeks of observation, the volumes of the tumors formed by subcutaneous injection of hOSE-CD164 cells was much greater than for SKOV3 cancer cells, and the injection of the hOSE-vector control and parental cells resulted in no tumor formation (Figure 3a and 3b). Furthermore, mice injected intraperitoneally with hOSE-CD164 cells but not hOSE-vector control cells developed peritoneal tumors, and these peritoneal tumors were significantly larger than those in mice injected with SKOV3 cells (Figure 3c). In addition, CD164 overexpression resulted in a greater formation of ascites in the hOSE-CD164 injected mice than the SKOV3 injected mice (Figure 3d).

### The knock-down of CD164 expression led to reduced tumorigenicity and enhanced the survival rate of cancer cell xenografted mice

**Figure 4:**
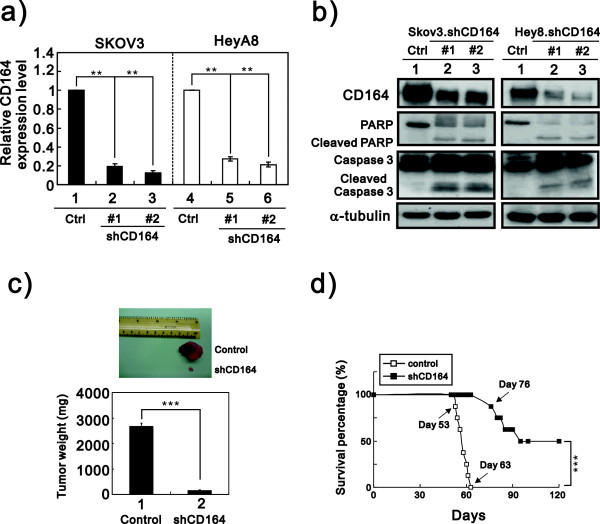
**CD164 downregulation rescues the effects of CD164 overexpression on tumorigenicity in nude mice. a)** Total RNAs were extracted from SKOV3-shCD164, HeyA8-shCD164 and respective control cells for the qRT-PCR analysis. Quantitative analysis of CD164 transcript levels relative to GAPDH was calculated and compared with SKOV3 or HeyA8 control cells. **b)** Doxycycline-inducible SKOV3-shCD164 and HeyA8-shCD164 cells were treated with 5 μg/ml doxycycline. After 48 hours, the cells were harvested and subjected to immunoblot analysis with antibody against CD164 protein, cleaved PARP and caspase 3 and its cleaved fragment. α-tubulin was used as a loading control. **c** and **d)** SKOV3-shCD164 cells and SKOV3-control cells were subcutaneously injected in the flanks of nude mice *(n* = 8). Nude mice were treated continuously with Dox and harvested at 8 weeks after Dox treatment. Tumor sizes and tumor weight **c)** were measured and analyzed, and Kaplan-Meier survival curves were recorded **d)**.

To investigate the functional roles of CD164 in tumorigenicity, we used shRNA targeting CD164 gene expression to investigate whether the downregulation of CD164 in ovarian cells could inhibit tumor growth and increase the survival time of xenografted mice. First, we established two viral targeted CD164 shRNA constructs with a doxycycline-inducible promoter, selected for successfully transfected cells using puromycin, and induced the expression of the shRNA constructs with doxycycline for 48 hours. We analyzed the downregulation efficiency of CD164 abundance in SKOV3 and HeyA8 cells by real-time PCR and immunoblotting analysis (Additional file 1: Figure S1 and Figure 4a and b). Next, we examined the activation of apoptosis by measuring the cleavage abundance of caspase-3 and PARP-1 proteins in the shCD164-SKOV3 and shCD164-HeyA8 cells, and our data demonstrated that the loss of CD164 induced cellular apoptosis is these two cell lines (Figure 4b). We further examined the antitumor effect of the downregulation of the CD164 protein in nude mice bearing shCD164-SKOV3 tumors. Six weeks after the subcutaneous injection of shCD164-SKOV3 and SKOV3-control cells, the mice were sacrificed, and the tumor growth was assessed by tumor volume. The volume of the tumors in the SKOV3-shCD164 groups was significantly decreased compared with the SKOV3-control cells (>90% reduction, n = 8, *p < 0.001*) (Figure 4c). On the other hand, the Kaplan-Meier survival curves showed that the SKOV3-shCD164 group had a significantly longer survival rate than the control group in the peritoneal injection model (n = 8, *p < 0.001*) (Figure 4d). The first death occurred at day 53 and 76, respectively, in the SKOV3-control and shCD164 groups. These data demonstrated that the downregulation of CD164 gene expression in ovarian cancer cells induced cellular apoptosis and reduced tumor growth to increase the survival time of xenografted mice.

### CD164 overexpression upregulates the SDF-1α/CXCR4 complex and activates downstream PI3K/Akt signaling

**Figure 5:**
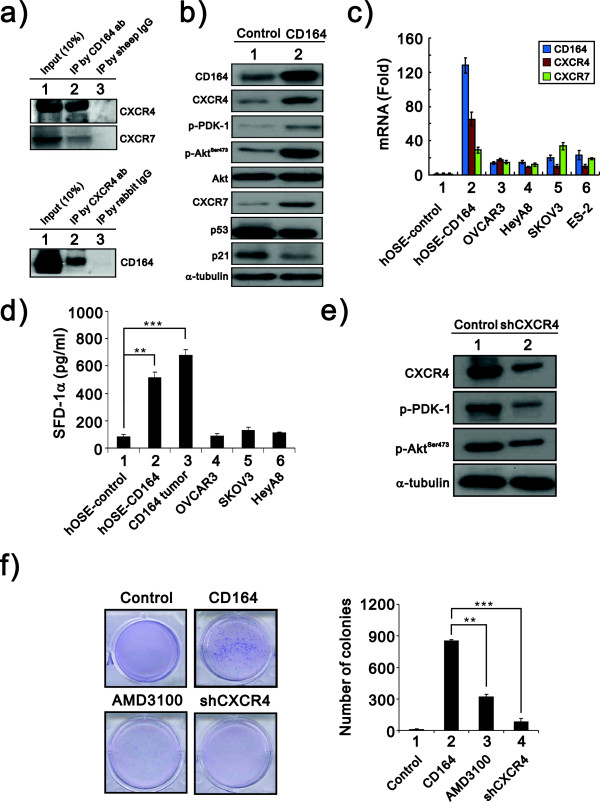
**CD164 overexpression upregulates the SDF1α /CXCR4 axis to activate downstream PI3K/Akt signaling pathway. a**-**b)** Cell lysates from hOSE-CD164 and hOSE-vector cells were subjected to **a)** immunoprecipitation with antibody against CXCR4 and immunoblot analysis with antibody against CD164 and a control antibody (upper) or immunoprecipitation with antibody against CD164 and immunoblot analysis with antibodies against CXCR4 and CXCR7 and a control antibody (bottom); **b)** immunoblot analysis with antibodies against CD164, CXCR4, pPDK1, pAkt and its phosphorylated form pAkt^Ser-473^, CXCR7, p53 and p21. α-tubulin was used as a loading control. **c)** Quantitative mRNA analysis of CD164, CXCR4 and CXCR7 in hOSE-CD164 cells and indicated ovarian cells was performed by qRT-PCR, and mRNA levels were normalized with individual GAPDH mRNA. Fold changes of specific mRNA expression were compared with that of hOSE control cells. **d)** The amount of SDF1α in culture medium from hOSE-CD164 cells and indicated ovarian cells was measured using an ELISA analysis. **e)** The hOSE-CD164 cells showed downregulated CXCR4 expression and were subjected to immunoblot analysis with antibodies against CXCR4, pPDK1 and pAkt^Ser-473^. α-tubulin was used as a loading control. **f)** For the colony formation analysis, the hOSE-CD164 cells were treated with the CXCR4 antagonist AMD3100 or CXCR4 was downregulated using shRNA. Quantitation of anchorage-independent growth of indicated conditioned cells was performed using anchorage independent assay. The presented data are the means of three experiments (means ± S.D.; n = 3).

The PI3 kinase/Akt pathway is commonly dysregulated in human cancers and functions in such processes as proliferation, survival and motility [21-23]. CXCR4, the upstream molecule of the PI3 kinase/Akt pathway, has been shown to directly or indirectly regulate tumor growth [20]. Furthermore, recent reports have indicated that CD164 interacts with CXCR4 and might regulate the pathway downstream of CXCR4 [11]. Our co-immunoprecipitation analysis showed that CD164 did complex with CXCR4 and CXCR7 (Figure 5a). We further addressed whether the SDF1α/CXCR4 axis is involved in CD164-induced ovarian tumor growth. The protein level of CXCR4 was induced, and its downstream signaling molecules, pPDK1 and pAkt^Ser473^, were activated in CD164 overexpressing hOSE cells compared with control cells (Figure 5b). Furthermore, our data showed that CD164 induced CXCR7 expression, another SDF-1α receptor, and reduced p53 and its target p21 expression (Figure 5b). We also confirmed that CD164 had the ability to increase the CXCR4 and CXCR7 mRNA levels in some ovarian cancer cells (Figure 5c). Several studies have reported that SDF1α stimulation leads to the activation of CXCR4 by phosphorylation of the cytoplasmic domain of CXCR4 for the activation of its downstream signaling pathways, MAPK, PI3K and STAT [24,25]. Hence, we investigated whether CD164 also induced SDF-1α production, and our ELISA data revealed more SDF-1α production in hOSE-CD164 cells and the cells isolated from the peritoneal xenografted tumor cells than in the other cancer cell lines (Figure 5d). Next, we designed a shRNA construct to silence the endogenous CXCR4 expression in hOSE-CD164 cells and found a decrease in the levels of pPDK1 and pAkt^Ser-473^ (Figure 5e). Finally, we observed a selective CXCR4 antagonist, AMD3100, and the silencing of CXCR4 expression in hOSE-CD164 cells counteracted the inductive effect of CD164 overexpression on colony number and anchorage independent growth (Figure 5f). Taken together, our data indicated that CD164 induced the expression of SDF-1α and CXCR4 to activate the SDF-1α/CXCR4 signaling pathway, causing tumorigenesis.

### Nuclear CD164 enhanced the promoter activity of the CXCR4 gene

**Figure 6:**
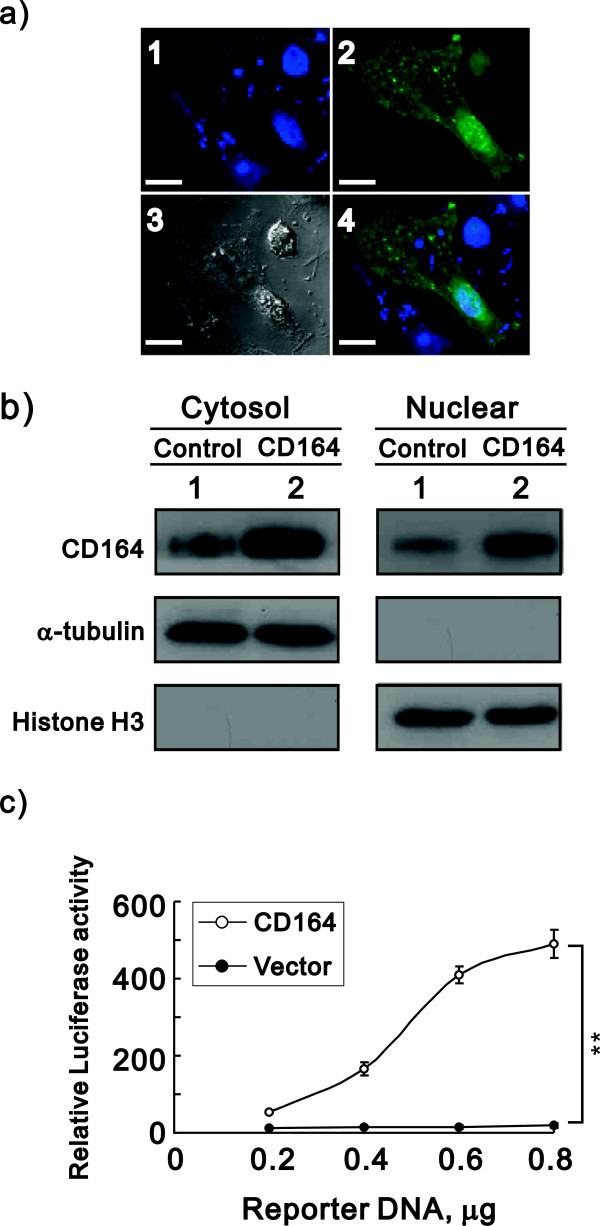
**Nuclear CD164 upregulates CXCR4 promoter activity. a)** Representative hOSE cells transiently transfected with EGFP–tagged CD164 constructs are shown. Approximately 100 transfected cells were examined to evaluate the intracellular distribution of each of the EGFP fusion proteins under a confocal microscope. Nuclei were stained with TOTO-3. Bar = 20 μm. **b)** Fractionated cytosolic and nuclear lysates from hOSE-CD164 and hOSE-vector cells were subjected to immunoblot analysis with antibody against CD164. α-tubulin was used as a loading control. **c)** Indicated amount of CXCR4 reporter DNAs were transiently transfected into hOSE-CD164 and hOSE-vector cells. After 36 hours, the luciferase activity in the transfected cell extracts was determined and the fold induction of the activity was estimated relative to that of cells transfected with the control vector in hOSE-vector cells. The presented data are the means of three experiments (means ± S.D.; n = 3).

Based on our tissue array data, which showed that CD164 proteins are located in the nucleus, we attempted to address whether the CD164 protein can directly activate the SDF-1α/CXCR4 axis via its function in the nucleus. We transiently transfected the CD164-EGFP vector into hOSE cells and observed, via a confocal microscope, that a proportion of the CD164 proteins were localized to the nucleus (Figure 6a). We further fractionated the lysates into cytoplasmic and nuclear fractions and reconfirmed that a proportion of the CD164 proteins were localized in the nucleus (Figure 6b). We examined the possibility that nuclear CD164 modulates the SDF-1α/CXCR4 axis via the enhancement of the CXCR4 promoter activity using the dual-luciferase reporter assay. The results revealed that CD164 enhanced the CXCR4 promoter activity in a dose-dependent manner in hOSE cells (Figure 6c).

### CD164 induces stem cell-specific transcription factors and form spheres

**Figure 7:**
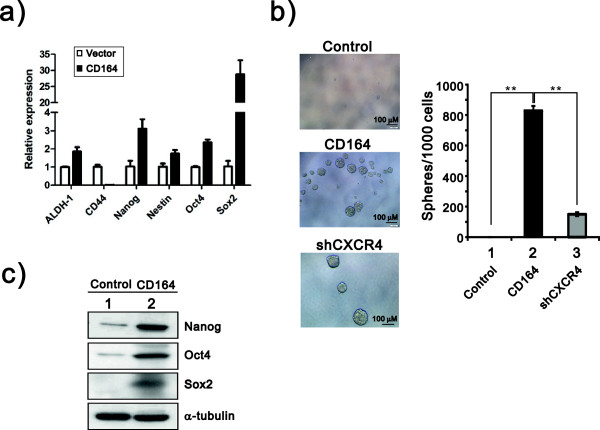
**The effects of CD164 on stem cell marker and sphere formation. a)** A quantitative mRNA analysis of ALDH-1, CD44, Nanog, Nestin, Oct4 and Sox2 in hOSE and hOSE-CD164 cells was performed by qRT-PCR, and mRNA levels are expressed relative to individual GAPDH mRNA. The presented data are the means of three experiments (means ± S.D.; n = 3). **b)** Spheroid formation of hOSE and hOSE-CD164 and shCXCR4 cells after culture in ultralow plates for 7 days. **p < 0.01. **c)** Western blot analysis of hOSE and hOSE.CD164 cell lysates were subject to against antibodies for CSC markers, including Nanog, Oct4 and Sox-2. α-tubulin was used as a loading control.

Cancer stem cells (CSCs) have been proposed to initiate and maintain tumor growth, and CD133 is a well-defined cancer stem cell marker [26,27]. A recent report indicated that the OSE expresses CD133 and other CSC markers, and malignant transformation can be induced in the OSE [28]. CXCR4 is highly expressed in various types of adult stem cells and CD133^+ high^ CSCs [29]. The SDF-1α/CXCR4 axis plays an important role in tumor progression and metastasis and indicates poor prognosis in ovarian cancer patients [30]. CD164 has been proven to be highly expressed in hematopoietic stem cells and other stem cells [11,31] and to be involved in some instances of cancer cell metastasis to other sites [14,15]. Our results demonstrated that CD164 also has important roles in the regulation of some stem cell specific transcriptional factors, such as Nanog, Oct4 and Sox-2 (Figure 7a). To investigate whether CD164 was involved in regulating the characteristics of CSCs, we determined the self-renewal capability of CD164-hOSE by an ultralow plate system. The sphere formation results showed that CD164 had sphere formation potential and knockdown of CXCR4 in CD164-hOSE significantly suppressed its ability to form spheres (Figure 7b). Furthermore, we double checked some important CSC markers, such as Nanog, Oct4 and Sox-2, whether induced by overexpression of CD164 in hOSE cells (Figure 7c). The induction of Nanog, Oct4 and Sox-2 proteins was consistent with their genes by CD164 (Figure 7a and c). Our findings suggest that CD164 may be involved in the self-renewal of CSCs through well-known markers and CXCR4. Therefore, CD164 overexpression might induce the OSE cells to become CSCs by activating the SDF-1α/CXCR4 axis, and CD164 could be a new marker for CSCs.

## Discussion

Previous studies have demonstrated the inhibition of cell proliferation by CD164 knock-down in colon cancer cells [15]. In this study, we explored the functional roles of CD164 in tumorigenesis using genetic loss-of-function and gain-of-function approaches. Overexpressing CD164 in hOSE cells may have the ability to induce tumor cell growth, proliferation, migration and self-renewal mediated through the induction of SDF-1α and CXCR4, which activates the SDF-1α/CXCR4 signaling pathway. An important result from the present study was that the nuclear localization of CD164 induced CXCR4 gene expression through the enhancement of CXCR4 promoter activity, suggesting that CD164 might be a potential transcription factor. The detailed mechanism of CD164 for this regulatory role needs to be further investigated. However, the effects of altering the abundance of CD164 on the modulation of cell survival and proliferation mediated through the SDF-1α/CXCR4 (or SDF-1α/CXCR4/CXCR7) axis and some stem cell specific transcriptional factors, such as Nanog, Oct4 and Sox-2, in ovarian epithelial cells was supported by similar mechanisms in the metastasis of CSCs and the trafficking of normal stem cells and provided promising results. In addition to above mentioned regulated genes by CD164, our mRNA microarray data also demonstrated that at least thousands of target genes were positively or negatively regulated in the hOSE-CD164 cells (data not shown). Hence, the tumorigenic capacity of CD164 might be mediated through its newly defined transcription factor function in multiple targets, including p53-dependent pathway, consistently that ovarian carcinogenesis is identified by multiple genetic alterations, combined transduction of mutant p53-KrasV12-AKT-c-myc, using hOSE tumorigenesis model [32].

The SDF-1α/CXCR4 axis plays critical roles in many physiological processes that involve cell migration and cell fate decisions, ranging from stem cell homing, angiogenesis and neuronal development to immune cell trafficking [20,33-36]. The predominant SDF-1α receptor is believed to be CXCR4, and signaling through CXCR4 alters the ability of cancer cell lines to adhere to the endothelium and invade through the extracellular matrix components, such as MMP-9, in the bone marrow [37,38]. Another highly important function of the SDF-1α/CXCR4 axis is related to tissue repair and regeneration [39]. CD164 associates with the chemokine receptor CXCR4, possibly as a co-receptor for SDF-1α, which is a likely mechanism by which CD164 influences migration and myotube formation and modulates the SDF-1α-mediated migration of umbilical cord blood CD133^+^ cells [11]. In addition, CD164 is most likely involved in the regulation of cell adhesion and proliferation via specific intercellular recognition [14]. In addition to CXCR4 as a receptor for SDF-1α, CXCR7 is another receptor and it can work alone or heterodimerize with CXCR4 to modulate, positively or negatively, CXCR4 signaling from SDF-1α [40,41]. The functional roles of CXCR7 are identified as CXCR4 does in many cancers, including prostate cancers [42,43]. Hence, our proposed the SDF-1α/CXCR4 axis by CD164 might be modified as the SDF-1α/CXCR4/CXCR7 axis because specific CXCR4 inhibitor or shCXCR4 not fully suppressed CD164-induced colony formation and sphere formation (Figure 5f and 7d). We had no further evidence of the CXCR7 gene regulation as CD164-directed CXCR4 promoter activity (Figure 6c). Hence, CXCR7 induction might be mediated through the direct effect from SDF-1α or the transactivation activity of CD164 in our case. It remains to be investigated in the future.

HIF-1α can bind to the CD164 promoter and induced CD164 gene expression; hence, hypoxia induction also leads to the upregulation of the CD164 gene expression via the transcription factor function of HIF-1α [44]. In addition to the induction of CD164 gene expression, HIF-1α enhances the expression and function of CXCR4, CXCR7 and SDF-1α on the surface of normal and malignant cells [43,45]. SDF-1α also enhances the expression of CD164 mRNA and alters the expression of the CD164 protein in prostate cancer cell lines [14]. In prostate carcinomas, not only the HIF-1α/AP-1 complex but also intracellular Zn^2+^ are involved in the induction of CD164 gene expression [44,46]. Combined with the results of the present study, we propose one positive-feedback working model in which environmental cues, such as hypoxia in tumorigenesis, induce CD164 overexpression and increase the components of the SDF-1α/CXCR4 axis to activate its downstream signaling pathways for cell proliferation, migration, invasion and self-renewal in ovary cancer cells. A recent study suggests that incorporating an inhibitor of CXCR4 into drug protocols for ovarian cancer may improve outcomes for patients with this disease [47]. Consequently, strategies aimed at modulating the SDF-1α/CXCR4 axis via the regulation of CD164 abundance could have important clinical applications in both tissue engineering and in clinical hematology and oncology for the inhibition of the proliferation and metastasis of CSCs.

## Materials and methods

### Cell lines and reagents

The human ovarian surface epithelial (hOSE) cell line OSE10, a gift from Professor Hidetaka Katabuchi (Kumamoto University, Japan) [48], was maintained in MCDB105 medium (Sigma Chemical Co., St. Louis, MO, USA) supplemented with 10% FBS, 10 ng/ml EGF (Sigma) and 400 ng/ml hydrocortisone (Sigma). The HEYA8, OVCAR3, ES-2 and SKOV3 human ovarian cancer cell lines were from the American type Culture Collection (Rockville, MD, USA) and were cultured with 10% fetal calf serum, 2 mM L-glutamine, 100 U/mL penicillin and 100 mg/mL streptomycin at 37°C in a humidified atmosphere consisting of 5% CO_2._ The CD164 polyclonal antibody (clone 502021) was purchased from R&D systems (Oxford, UK). CXCR4 antibody (clone ab2074) was obtained from Abcam (Cambridge, United Kingdom). CXCR7 antibody (clone C1C2), Nanog antibody (GTX627421) and Sox2 antibody (GTX62242) were purchased from Genetex (Taipei, Taiwan, ROC). Akt antibody, the phospho-Akt antibody, histone H3 antibody and Oct4 antibody were purchased from Cell Signaling Technology (Danvers, MA, USA). p53 (DO-1) and p21 (C-19) antibodies were purchased from Santa Cruz Biotechnology (Santa Cruz, CA, USA).

### Lentiviral infections

The lentiviral CD164 shRNA constructs were purchased from the National RNAi Core Facility in Academic Sinica, Taipei, Taiwan. The target sequences of these shRNAs are described in Table 2. Lentiviruses were produced by cotransfecting the shRNA-expression vectors pMD2.G and psPAX2 into 293 T cells using calcium phosphate. Viral supernatants were harvested and used to infect SKOV3 and HeyA8 cells with 8 μg/mL polybrene. Cells were selected using 2 μg/mL puromycin. CD164 overexpressing cells were established by infection with the lentivirus-expressing pWPXL-vector with the human CD164 coding sequence.

**Table 2:** shRNA sequence list

**shRNA name**	**Sequence (5′ to3′)**
CD164 shRNA 1	GCTATTGTTCACATAACTCAA
CD164 shRNA 2	CGTGACGACTTTAGCGCCCAT
CXCR4 shRNA1	AGATAACTACACCGAGGAAAT
CXCR4 shRNA2	TCCTGTCCTGCTATTGCATTA

### Isolation of total RNA and analysis of quantitative real-time PCR

Total mRNA was extracted using the TRIzol reagent (Invitrogen, Carlsbad, CA, USA), and reverse transcription was performed using an RT–PCR kit (Invitrogen). Analysis of quantitative real-time PCR (qPCR) was performed on an ABI 7500 instrument (Applied Biosystems, Foster City, CA, USA) using the SYBR Green PCR Master Mix (Applied Biosystems). Each measurement was performed in triplicate. For each qPCR, a dissociation curve analysis was conducted, and GAPDH was applied as the internal housekeeping gene control. All PCR primers were listed in Table 3.

**Table 3:** Primer sequence list

**qPCR Primer name**	**Sequence (5′ to 3′)**
CD164-F	GGCACCAGAAACCTGTGAAG
CD164-R	TGTCGTGTTCCCCACTTGAC
CXCR4-F	GAACCCTGTTTCCGTGAAGA
CXCR4-R	CTTGTCCGTCATGCTTCTCA
CXCR7-F	CACAGCACAGCCAGGAAGG
CXCR7-R	GTTCCCTGGCTCTGAGTAGTCGA
ALDH1-F	TGGCTTATCAGCAGGAGTGT
ALDH1-R	GCAATTCACCCACACTGTTC
CD44-F	TCCCAGACGAAGACAGTCCCTGGAT
CD44-R	CACTGGGGTGGAATGTGTCTTGGTC
Nanog-F	GAAATCCCTTCCCTCGCCATC
Nanog-R	CTCAGTAGCAGACCCTTGTAAGC
Nestin-F	CTGCGGGCTACTGAAAAGTT
Nestin-R	AGGCTGAGGGACATCTTGAG
Oct4-F	TCAGGTTGGACTGGGCCTAGT
Oct4-R	GGAGGTTCCCTCTGAGTTGCTT
Sox2-F	GAGGGCTGGACTGCGAACT
Sox2-R	TTTGCACCCCTCCCAATTC
GAPDH-F	ACCCACTCCTCCACCTTGACG
GAPDH-R	TCTCTTCCTCTTGTGCTCTTG

### Immunoblot assay

The cell lysates were prepared in RIPA lysis buffer (150 mM NaCl, 1% NP40, 0.5% DOC, 50 mM Tris–HCl at pH 8, 0.1% SDS, 10% glycerol, 5 mM EDTA, 20 mM NaF and 1 mM Na_3_VO_4_) supplemented with 1 mg/ml each of pepstatin, leupeptin, and aprotinin and 200 mg/ml phenyl-methylsulfonyl-fluoride. The lysates were separated by sodium dodecyl sulfate–polyacrylamide gel electrophoresis, transferred onto polyvinylidine difluoride membranes (Millipore, USA) and detected by using the primary antibodies indicated above.

### Patients and tissue microarray

Surgical resection of 97 tumor samples from primary ovarian cancer patients and normal ovary samples from a commercial ovarian cancer tissue array (OVC1021, Pantomics, Inc.) were studied.

### Immunohistochemistry

The tumor tissues were dissected from intraperitoneally injected animals. The tissues were processed by fixation in 4% buffered formalin and then embedment in paraffin wax. The sections (5 μm) were stained with the hematoxylin and eosin stained for histopathologic analysis. After the sections were dried overnight at 37°C, they were deparaffinized with xylene. The sections were treated with an antigen retrieval solution (Target Retrieval, Dakocytomation, Carpinteria, CA, USA) at 95°C for 15 minutes and incubated overnight at 4°C with a rabbit monoclonal antihuman CXCR4 antibody (Abcam) and polyclonal sheep antihuman CD164 antibody (R&D), both at a dilution of 1:1000. The next day, the sections were incubated with streptavidin linked to horseradish peroxidase (Dako Corp., Carpinteria, CA, USA) and then with a secondary mouse anti-immunoglobulin antibody linked to biotin; they were then developed with diamino-benzamidine (DAB) and counterstained with hematoxylin.

### Animals

All of the animal experiments were approved (IACUC NO.12-094) and conducted under the guidance of the Institutional Animal Care and Use Committee (accredited by the Association for Assessment and Accreditation of Laboratory Animal Care International), National Defense Medical Center, Taipei, Taiwan. A total of 4x10^6^ cells in 0.1 mL of PBS were injected subcutaneously or intraperitoneally (n = 8 for each group) into female athymic nude mice of 7–8 weeks of age, and tumor growth was followed for 7–8 weeks. After sacrifice, the solid tumors and ascites were quantified and assayed. For the survival study, nude mice were intraperitoneally injected with 4x10^6^ cells and the time of death of each animal was recorded (n = 8 for each group).

### Cell proliferation assay

hOSE cells were seeded at a density of 1x10^4^ cells/ml (100 μl/well) into 96-well microplates with complete medium and were cultured for 24 hr. After 24 hr of incubation, the cell proliferation was assessed by the BrdU assay method. The absorbance at 450 nm was determined using a microplate reader.

### Soft agar colony formation assay

Bottom agar (0.7%) was plated onto six-well plates and 10,000 cells were plated in triplicate, incubated at 37°C overnight and covered with 0.2 ml DMEM media. Colonies were allowed to form over 2–3 weeks and were then stained by crystal violet for counting. Images were taken at 10× magnification.

### ELISA

Quantitative ELISA (Quantikine, R&D Systems) was used to determine the level of SDF-1α in the culture medium. The samples and standards were pipetted into wells pre-coated with a monoclonal antibody specific for SDF-1α. After washing, an enzyme-linked polyclonal antibody specific for SDF-1α was added. The optical density was measured using a microplate reader set to 450 nm with a correction at 540 nm.

### Coimmunoprecipitation

Cells were lysed for 30 min at 4°C in immunoprecipitation (IP) buffer (10 mM Tris–HCl, pH 7.4, 150 mM NaCl, 1 mM EDTA, 1 mM EGTA, 1% Triton X-100, 0.5% NP-40, 0.75 mM MTA, 0.2 mM Na_3_VO_3_, and 0.2 mM PMSF). Lysates were incubated with CD164 and CXCR4 antibody overnight at 4°C, then pre-treated protein-G Magnetic Beads (Millipore) incubated 2 hours at 4°C. Beads were wash and resuspended in sample buffer and samples subjected to SDS-PAGE/Western blotting. Blots were revealed with sheep anti-CD164 (1:1000), rabbit anti-CXCR4 (1:1000), CXCR7 (1:500), HRP-conjugated anti-sheep (1:1000) or anti-rabbit (1:1000) antibodies were used as secondary antibodies. Chemiluminescence was detected by ECL (Pierce, Rockford, IL, USA).

### Immunofluorescence

The cells were grown on coverslips, fixed with a 4% paraformaldehyde-PBS solution and permeabilized with 0.2% Triton X-100 in PBS. Following fixation, the cells were blocked with 5% bovine serum albumin (BSA) for 1 hour and incubated at 4°C for 18 hours with a primary antibody (1:100) in 5% BSA in PBS. The cells were then incubated with a secondary antibody (1:200) at room temperature for 45 minutes in stock buffer. The photographs were taken with the ×63/oil 1.4 DIC objective of a Zeiss LSM510 META confocal laser-scanning microscope (Axiophot 2, ZEISS) (Carl Zeiss, Jena, Germany).

### Luciferase reporter assay

To determine the chemokine promoter activity, hOSE cells were cultured to 80% confluency in six-well plates and transfected in each well (24-well plate) with jetPEI (Polyplus-transfection, France), according to the manufacturer’s protocol. The total DNA for transfection was adjusted to 1.0 μg by the addition of the empty vector. The luciferase assays were performed with the Promega Luciferase Assay Kit (Madison, WI, USA), and the measurements were expressed numerically as relative light units (RLU). Luciferase activities are given as the means and standard deviations of two transfected sets of cells. The results shown are representative of at least three independent experiments.

### Sphere formation assay

Tumor spheres were generated in serum-free sphere medium consisting of Dulbecco’s modified Eagle’s medium and supplemented with 20 ng/mL epidermal growth factor (EGF; Sigma) and 20 ng/mL basic fibroblast growth factor (bFGF; Sigma). hOSE and hOSE.CD164 cells were seeding 1000 cells (10 cells per well) in 96-well ultra-low attachment plates in 100 μL of serum free medium and cultured for up to 7 days. The number of spheres was evaluated after 4 days.

### Statistical analysis

Data from different experiments were presented as the means ± SEM and were analyzed by the two-tailed Student’s t test. P < 0.01 was considered significant. The data were the means ± standard deviations (SD) for at least three independent experiments. Statistical significance was assessed using a one-way ANOVA and Student’s t-test. * indicates P < 0.05, **P < 0.01 and *** P < 0.001, respectively, when compared with the control.

## Abbreviations

EOC: Epithelial ovarian cancer; hOSE: Human ovarian surface epithelial; shRNA: Short hairpin RNA; dox: Doxycycline; RT: Reverse transcription; qPCR: Quantitative polymerase chain reaction; RLU: Relative light units; EGFP: Enhanced Green Fluorecence Protein; CXCR: C-X-C chemokine receptor type 4; SDF-1: Stromal cell-derived factor 1; GAPDH: Glyceraldehyde3-phosphate dehydrogenase; MMP9: Matrix metallopeptidase 9; PI3K: Phosphatidylinositide 3-kinases; AKT: Protein Kinase B; p-AKTSer473: Phosphorylated Akt at serine 473; Caspase-3: Cysteine-aspartic proteases; BAX: Bcl-2-associated x protein; Bcl-2: B-cell-lymphoma-2; PARP: Poly(ADP-ribose) polymerase; HIF1α: Hypoxia-inducible factor 1-alpha; Sox-2: SRY (sex determining region Y)-box 2; Oct4: Octamer-binding transcription factor 4.

## Competing interests

The authors declare that they have no competing interests.

## Authors’ contributions

AFH carried out the culture, qRT-PCR, ELISA, histoimmunostaining analysis, xenograft model and drafted the manuscript. MWC participated in the immunostaining analysis and xenograft model. CLK carried out the culture, and PCR examination. SMH drafted the manuscript. HCL participated in cancer stem cell marker qRT-PCR analysis. JYHC conceived of the study, and participated in its design and coordination and helped to draft the manuscript. All authors read and approved the final manuscript.

## Supplementary Material

Additional file 1: Figure S1 Inducible downregulation of CD164 in Hey8 and Skov3 cells. SKOV3-shCD164 and HeyA8-shCD164 cells were treated with 0, 1, 2 and 5 μg/ml of Dox for 48 hours, and then, CD164 protein expression was analyzed by immunoblot with an antibody against the CD164 protein. α-tubulin was used as a loading control. Click here for file
