# Supplementary material for: CD164 regulates the tumorigenesis of ovarian surface epithelial cells through the SDF-1α/CXCR4 axis
Source: Mol Cancer. 2013 Oct 5;12:115. doi: 10.1186/1476-4598-12-115 (PMC4015273; doi:10.1186/1476-4598-12-115)
Supplement: Additional file 1: Figure S1 — Inducible downregulation of CD164 in Hey8 and Skov3 cells. SKOV3-shCD164 and HeyA8-shCD164 cells were treated with 0, 1, 2 and 5 μg/ml of Dox for 48 hours, and then, CD164 protein expression was analyzed by immunoblot with an antibody against the CD164 protein. α-tubulin was used as a loading control. [file 1476-4598-12-115-S1.pdf]

Huang et al

# Figure S1

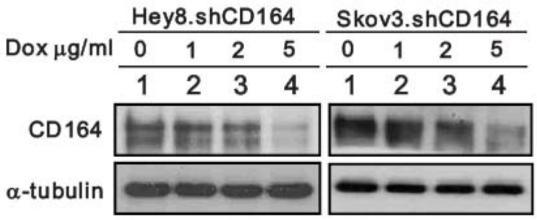


**Figure S1 Inducible downregulation of CD164 in Hey8 and Skov3 cells.** SKOV3-shCD164 and HeyA8-shCD164 cells were treated with 0, 1, 2 and 5 μg/ml of Dox for 48 hours, and then, CD164 protein expression was analyzed by immunoblot with an antibody against the CD164 protein. α-tubulin was used as a loading control.
